# Automatic Tunnel Crack Detection Based on U-Net and a Convolutional Neural Network with Alternately Updated Clique

**DOI:** 10.3390/s20030717

**Published:** 2020-01-28

**Authors:** Gang Li, Biao Ma, Shuanhai He, Xueli Ren, Qiangwei Liu

**Affiliations:** 1 School of Electronic and Control Engineering, Chang’an University, Xi’an 710064, Shaanxi, China; 2018232026@chd.edu.cn (X.R.); 2018132015@chd.edu.cn (Q.L.); 2 Key Laboratory of Road Construction Technology and Equipment of MOE, Chang’an University, Xi’an 710064, Shaanxi, China; 3 Key Laboratory for Old Bridge Detection and Reinforcement Technology of Ministry of Transportation, Chang’an University, Xi’an 710064, Shaanxi, China; heshai@chd.edu.cn

**Keywords:** tunnel crack, U-net, CliqueNet, crack measurement

## Abstract

Regular crack inspection of tunnels is essential to guarantee their safe operation. At present, the manual detection method is time-consuming, subjective and even dangerous, while the automatic detection method is relatively inaccurate. Detecting tunnel cracks is a challenging task since cracks are tiny, and there are many noise patterns in the tunnel images. This study proposes a deep learning algorithm based on U-Net and a convolutional neural network with alternately updated clique (CliqueNet), called U-CliqueNet, to separate cracks from background in the tunnel images. A consumer-grade DSC-WX700 camera (SONY, Wuxi, China) was used to collect 200 original images, then cracks are manually marked and divided into sub-images with a resolution of 496 × 496 pixels. A total of 60,000 sub-images were obtained in the dataset of tunnel cracks, among which 50,000 were used for training and 10,000 were used for testing. The proposed framework conducted training and testing on this dataset, the mean pixel accuracy (MPA), mean intersection over union (MIoU), precision and F1-score are 92.25%, 86.96%, 86.32% and 83.40%, respectively. We compared the U-CliqueNet with fully convolutional networks (FCN), U-net, Encoder–decoder network (SegNet) and the multi-scale fusion crack detection (MFCD) algorithm using hypothesis testing, and it’s proved that the MIoU predicted by U-CliqueNet was significantly higher than that of the other four algorithms. The area, length and mean width of cracks can be calculated, and the relative error between the detected mean crack width and the actual mean crack width ranges from −11.20% to 18.57%. The results show that this framework can be used for fast and accurate crack semantic segmentation of tunnel images.

## 1. Introduction

### 1.1. Motivation

At present, tunnel construction technology is becoming increasingly sophisticated, but how to maintain the tunnels is a problem troubling the society [1]. The safety threat of aging tunnels has been recognized as a growing national public concern, as most tunnels are used for a long time and need regular inspection and maintenance [2]. Cracks are the main factor affecting the service life of a tunnel. Traditional monitoring methods rely on manual crack detection [3], but the process is subjective and operators face very difficult or even dangerous conditions, such as dust conditions, insufficient light or toxic exposure [4]. Another reason is that it’s time-consuming and laborious to detect the cracks from thousands of pictures manually in front of the computer, so researchers are trying to develop techniques that can detect cracks automatically [5]. How to use the least personnel to continuously and automatically monitor the building has become an important research direction [6].

**Figure 1:**
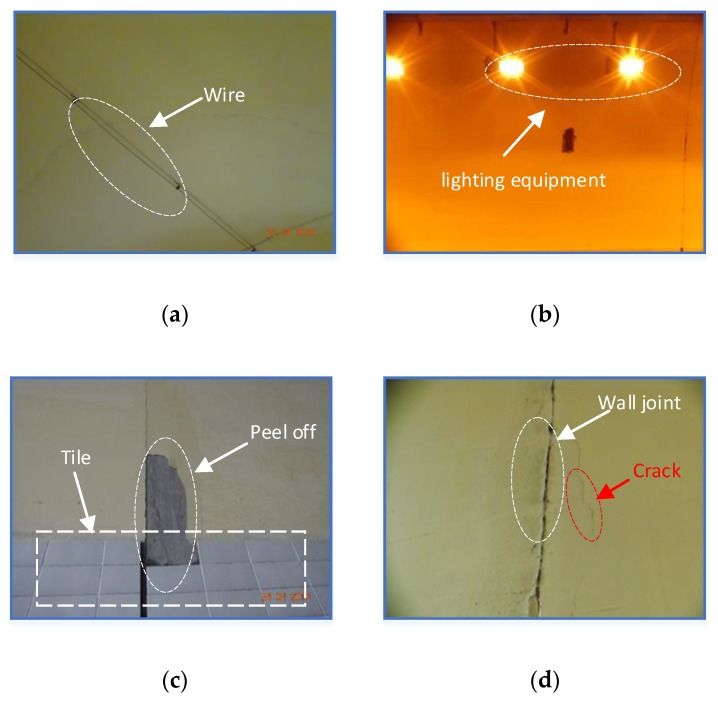
Some disturbing factors in the tunnel vault: (**a**) wire, (**b**) lighting equipment, (**c**) the calcimine peels off and tiles, (**d**) wall joint.

Crack detection technology has a long research history, but most of the methods developed are devoted to the detection of pavement cracks, in other words, there are few algorithms for tunnel crack detection. Due to the complex internal environment of the tunnel, most automatic crack detection methods are not accurate or effective enough, so it is a challenging task to detect the length and width of cracks in tunnel vaults and side walls automatically. In order to realize the study described in this paper, we built a tunnel image dataset by taking a large number of images in different tunnels. The camera we used was a consumer-grade DSC-WX700 camera (SONY, Wuxi, China), which was placed perpendicular to the wall and at about 50 cm from the wall. More details of the data acquisition method are presented in Section 3.1. Figure 1 shows some of the images, including many of the complex elements inside the tunnel. For example, Figure 1a,b show the presence of wires and lighting equipment in the tunnel vault, while Figure 1c,d, demonstrate the side wall with tiles falling off and the patchwork joint of the wall which is very similar to a crack. In addition, the chromatic aberration of images taken in different tunnels is very large, which increases the difficulty of tunnel crack identification.

### 1.2. Related Works

Edge detection methods are widely adopted in early crack detection. One of them is the Laplacian of the Gaussian algorithm [7,8]. A robotic crack inspection and mapping (ROCIM) system is proposed to provide an overall solution to the bridge deck crack inspection. Edge detection algorithms such as Roberts, Prewitt, Sobel, Laplacian of Gaussian, Butterworth, and Gaussian were used to detect cracks in 3420 concrete sub-images [9]. These methods can accurately detect 53–79% of cracked pixels, but lead to residual noise in the final binary image. In conclusion, the main drawback of edge detection methods is that it can only detect a group of disjoint crack patches, which often fail in low-contrast and high-clutter images.

Early unsupervised methods of crack detection [10] were normally based on threshold segmentation. These kinds of methods [11,12] have been widely studied due to their simplicity, but these methods are sensitive to noise, resulting in unreliable crack detection results, especially in the case of poor illumination and large visual clutter. Otsu’s threshold is adopted [13] to detect cracks in 2D images. It has high accuracy but low precision. The application of image threshold segmentation or edge detection algorithms to the detection of road cracks has achieved good results, but the its application to the detection of tunnel cracks is completely useless. Li et al. recently proposed an unsupervised multi-scale fusion crack detection (MFCD) algorithm, which combined the advantages of large-scale detection and small-scale detection, and achieved good results in a public dataset of road cracks [14]. The road background is relatively simple, but examining even a common tunnel will require a lot of electronic equipment and lighting equipment, so this method is completely useless in a tunnel context. 

In recent years, deep neural networks have made great progress in the field of image processing. They can make an algorithm automatically learn the target to be detected, which also makes it very adaptable to complex environments. CrackNet, an efficient architecture based on convolutional neural networks (CNNs), was proposed by Wang et al. to detect 3D asphalt surface cracks at a pixel-level, so it does not have any polling layers [15], and then CrackNet II was proposed, which has higher accuracy and a deeper architecture with more hidden layers but fewer parameters [16]. Zhang et al. trained a deep convolutional neural network with transfer learning to preclassify a pavement image into cracks, sealed cracks, and background regions [17]. Cha’s CNN has been a success, but this network can only be used for crack classification, not for crack segmentation [18].

CNNs achieve high detection accuracies whether an image or a sub-image contain cracks or not, Dorafshan et al. compared the performance of common edge detectors and deep convolutional neural networks (DCNNs) in the detection of concrete cracks, and their results showed that the DCNNs method had an important application prospect in the field of damage detection of concrete structures [9]. Many researchers began to make further improvement based on CNNs, including changing the structure of the convolutional neural network and combining other algorithm theories to better realize the prediction of cracks. Hui et al. proposed a modified fusion convolutional neural network, using three bypass stages to combine the multilevel features, that can divide a sub-image into three categories: crack elements, handwriting elements and background [19]. After classifying images by convolutional neural networks, Chen et al. processed the classification results with Naive Bayes, making the results more robust [20]. All these results indicate that CNNs have great research prospects, and many researchers have begun to modify the structure of CNNs to achieve better effects.

The previous convolutional neural network is used to classify cracks. Because the shape and color of cracks are relatively simple, many algorithms can effectively identify whether there are cracks in an image. However, with the introduction of fully convolutional networks (FCN) [21] and U-net [22], it is proved that spatial features can be obtained by transposed convolution layer after convolution and pooling, which can be extended and applied in the field of image segmentation. FCN (fully convolutional networks ) was adopted in the crack segmentation field by Yang et al. and it achieved an end-to-end prediction [23]. Huang et al. designed a two-stream FCN model, which can detect cracks and leakage of metro shield tunnels, respectively [24]. U-net was used for biomedical image segmentation, where it can be trained end-to-end from very few images and outperforms the prior best method. The U-net consists of a contracting path to capture context and a symmetric expanding path that enables precise localization. Liu et al. applied U-Net to detect cracks of concrete pavement under various conditions [25], and their results showed that this method is more accurate than FCN when the training set is small. 

A recent study developed a convolution neural network with alternately updated clique (CliqueNet), which has achieved state-of-the-art performance in the field of image classification [26]. There are both forward and backward connections between any two convolution layers in the same block, for each layer, it is both the input and output of any other layer. The results show that the algorithm can highlight the target features and weaken the influence of background and noises.

### 1.3. Contribution

CliqueNet has achieved good results in image classification because cliqueblock is used instead of an ordinary convolutional layer, while U-net reaches similar accuracy to FCN under the condition of fewer datasets. Therefore, based on the ideas of U-net and CliqueNet, this paper proposes a fused U-CliqueNet which can divide the cracks from the background more accurately and quickly. The contributions of this study are as follows:

1. A new deep learning network based on Clique-net and U-net called U-CliqueNet is proposed for semantic segmentation of tunnel cracks from images.
2. The proposed model integrates clique block and into U-net and adds an attention mechanism in the process of down-sampling, which makes it better than U-net in dealing with crack segmentation noises.
3. A tunnel crack dataset is established, including various cracks and disturbances. The proposed model is tested on this dataset, and the length and mean width of cracks can be calculated automatically.

## 2. Methodology

As described in Section 1.3, this paper uses some of the techniques in the CliqueNet to improve the U-net network, forming a new network named U-CliqueNet. In this part, we describe the proposed method in detail. First, in order to understand the proposed method more clearly, we give a brief introduction to U-net and CliqueNet. Then, we introduce the specific method of combining U-net with Cliquenet, that is, the model proposed in this paper.

### 2.1. Review of U-net and CliqueNet

**Figure 2:**
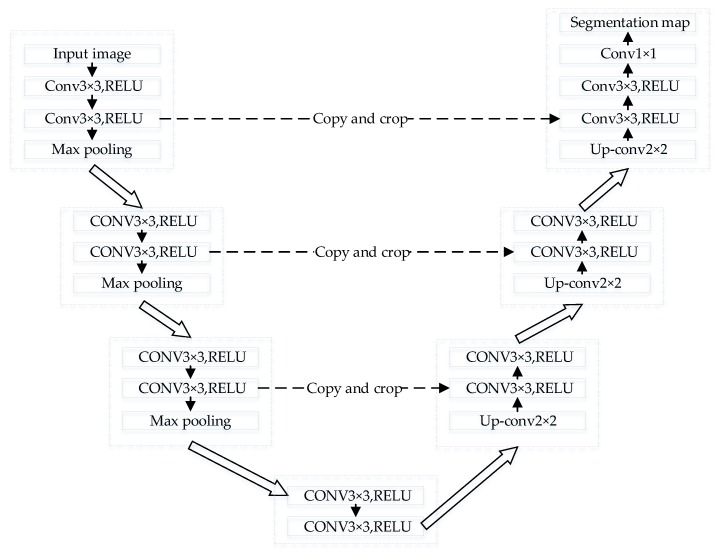
U-net architecture.

The architecture of the original U-net is illustrated in Figure 2. It consists of a symmetrical contraction path and an expansive path.

The contraction path uses the ordinary convolutional layer and pooling layer, while the expansion path uses deconvolution instead of the maximum pooling layer. In U-net, all convolutional layers are unpadded convolutions, each followed by a nonlinear activation function Rectified Linear Unit (ReLU) [27] and a 2×2 max pooling operation with steps of 2 for downsampling. After each deconvolution of the expansion path, the characteristics of the corresponding contraction path before pooling are merged with it. The copy and crop operation combines the high-resolution characteristics of the shrink path with the upsampling output, enabling the network to predict a more accurate output from the assembled information. At the last layer, the 1×1 convolution is adopted to map each feature vector to the required number of classes. It is worth mentioning that in the contraction path, the convolution operation after each downsampling can double the number of channels of the feature map, while in the expansion path, it is the opposite

**Figure 3:**
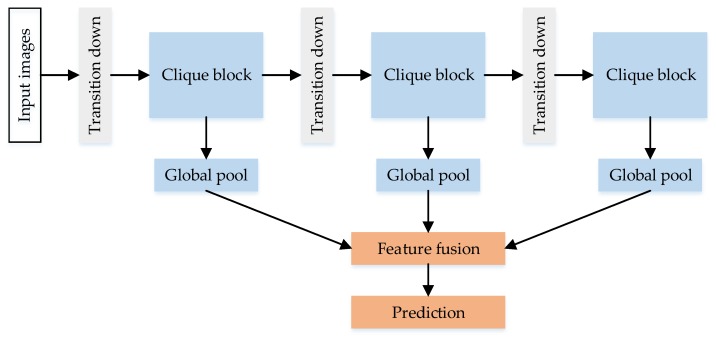
The original CliqueNet architecture.

The difference between the CliqueNet and the traditional convolutional neural network is that it proposes the clique block based on the DenseNet, and the output result is obtained by combining the output feature of each block, as shown in Figure 3. There are three clique blocks in CliqueNet, and the transition down layer between any two blocks uses a convolution and an average pooling to change map sizes.

**Figure 4:**
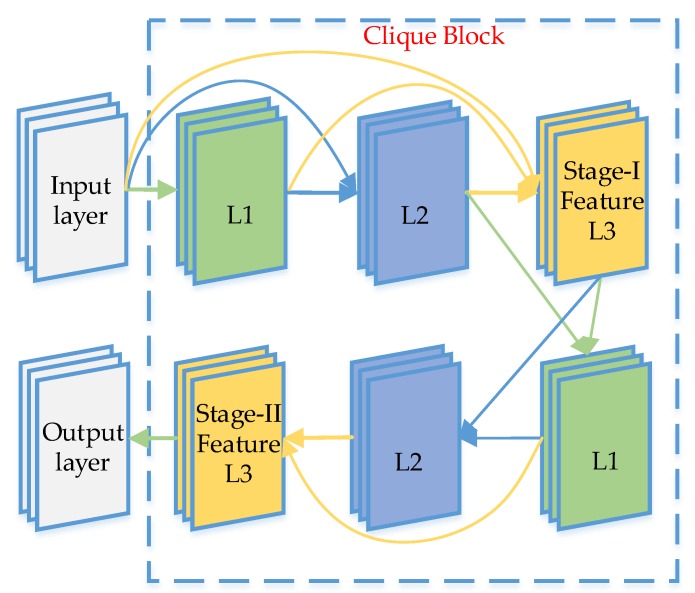
Clique block: The data entered from the previous block to the next layer will be looped through the block twice.

The clique block is the core of the CliqueNet. The architecture of a clique block with three layers is presented in Figure 4.

By linking all layers in the same block, it can acquire more information that is useful for crack classification. The method is to connect any two layers in the same block except for the input layer, in other words, every layer is both the input and output of any other layers in the Clique block. In addition, each convolutional layer will followed by a batch normalization and a ReLU operation. This kind of network can combine the information learned in the shallow layer with the information learned in the deep layer to predict the target more accurately

The data entered from the previous block to the next layer will be looped through the block twice, with the first looping feature called stage-I feature and the second looping feature called stage-II feature. First, the input layer ($X_{0}$) input data to this block, and it will be the input of all layers within the block, each updated layer is concatenated to update the next layer. At the second stage, the input of each layer is the most recent output of the other layers. According to this principle, the i−th (1≤i ≤4) layer in the first loop can be expressed by Equation (1), and the i−th (1≤i ≤4) layer in the second loop can be calculated by Equation (2):

(1) $$X_{i}^{\left(1\right)}=\sigma \left(W_{0i}⊗X_{0}+\sum_{l<i}W_{li}⊗X_{l}^{\left(1\right)}\right),$$

(2) $$X_{i}^{\left(2\right)}=\sigma \left(\underset{l<i}{\sum }W_{li}⊗X_{l}^{\left(2\right)}+\underset{m>i}{\sum }W_{mi}⊗X_{m}^{\left(1\right)}\right)$$

where ⊗ represents the convolution operation, $W_{ij}$ denotes the weights of parameters from $X_{i}$ to $X_{j}$, and σ is the RELU function to maintain non-linearity. In all clique blocks, each layer receives information from the other layers that were recently updated. The spatial attention mechanism is realized through the top-down refinement brought by each propagation. This repetitive feedback structure ensures maximum communication between all layers in the block.

The role of the first stage is mainly to initialize all the layers in each block, which will be further refined in the second stage. Due to the high computational complexity and model complexity of higher order propagation, we decided to use only the second stages. Considering that the features obtained in the second stage are more refined than those obtained in the first stage, we take the output of the last layer of the second stage as the output of each block. The results show that the characteristics of the second stage do improve the calculation results significantly. 

### 2.2. Overall Architecture of U-CliqueNet

**Figure 5:**
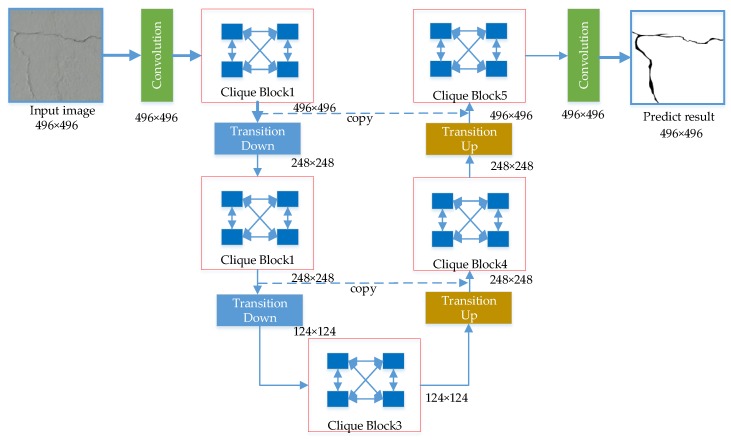
Overview of the proposed crack detection framework: U-CliqueNet.

In this section, the proposed model for the semantic segmentation of tunnel crack is described. Figure 5 demonstrates the overview of the proposed crack segmentation framework called U-CliqueNet, which consists of a contracting path and an expansive path like U-net. On the basis of U-net, the proposed model uses clique blocks to replace the ordinary convolutional layer and reduces the number of down sampling operations. The three main components of the proposed framework are clique block, transition down layer and transition up layer. The proposed model has five clique blocks with four layers in each block, and each layer contains 36 filters. There are two transition down layers and two transition up layers in the proposed networks, the output size of the final layer is the same with input image. In the down-sampling path, the features output by each block are not only transmitted to the next layer, but also preserved for feature fusion in the symmetric up-sampling process, so as to obtain better prediction results. The cropping is adopted in U-net due to the loss of border pixels in every convolution, but in our model, the padding operation is adopted in every convolution layer. For this reason, the scale of feature maps will not change, there is no crop operation.

Firstly, a sub-image with a resolution of 496×496×3 is put into the network. After the first convolution layer with 3×3 kernels, the image becomes a feature map of 496×496×96. Note that in our models, every convolution layer is padded by one pixel to maintain the same size of the feature maps.

The clique block concept will be detailed in Section 2.2, and the output size of clique block1 is 496×496×36. The transition down (TD) operation consists mainly of convolution layers and pooling layers; more details will be introduced in Section 2.2. The feature map size of the first transition down layer will be reduced by half due to the polling operation. The transition up (TU) layer has only the transposed convolution operation, the feature map size will be doubled after this layer. The purpose of copy path is to obtain more useful feature by combining shallow feature with deep feature, the channels are added behind copy path. Table 1 lists the output sizes and the operations for all convolution, transition down, and transposed convolution layers. Note that each “conv” operation shown in the table corresponds the sequence BN-ReLU-Convolution, and each “avp” represents an average pooling operation.

**Figure 6:**
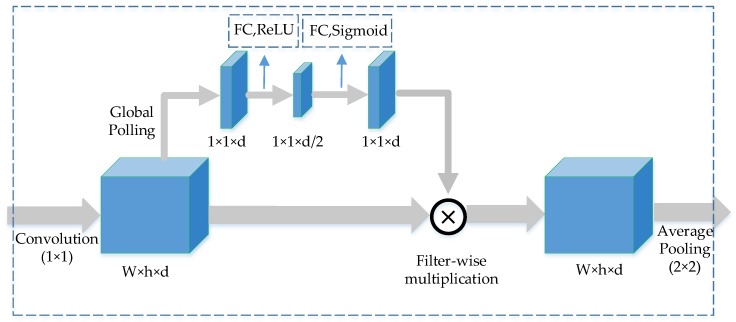
The transition down layer in down sampling process, it consists of convolution layer, fully connected layer and average pooling layer. W, h and d are width, height and depths of feature maps, respectively.

Unlike the traditional pooling layer, our downsampling layer introduces an attentional mechanism, as shown in Figure 6. This channel-wise attention mechanism is also used in CliqueNet, and the effectiveness of the attention mechanism is obtained through comparative experiments. First, the input feature reaches the first convolutional layer, and then it is divided into two outputs. The lower path is the main feature, while the upper path represents the weight of each channel. If the upper path weight is dotted with the lower path feature, the background can be reduced and useful features can be enhanced.

The filters are globally averaged after the convolution layers, and behind them are two fully connected layers. The first full connection layer reduces the data by half and is activated by RELU function, while the second fully connected layer doubles the data and is activated by sigmoid function. The feature in the upper path is scaled to [0, 1], which is multiplied by the features of the lower path in filter-wise. Average pooling is performed on the obtained result, and the final output of the transition down layer is obtained. This attention mechanism is only adopted in transition down layers to prevent the networks overfitting.

**Figure 7:**
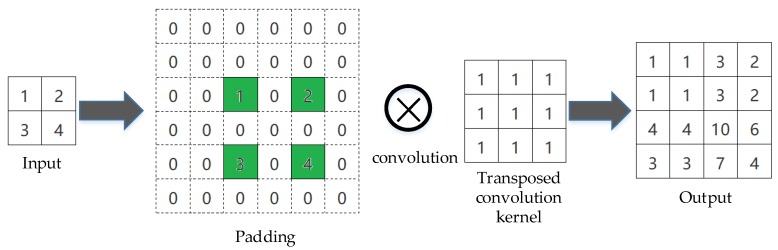
An example of transposed convolution operation.

Transposed convolution is the only operation in the transition up layer. Common convolution operation is widely used in convolutional neural network, while transposed convolution is rarely used. As Figure 7 describes, there is an example of a transposed convolutional layer with a specific stride and padding, which converts a coarse input feature map into a dense output feature map. The pixel value of 0 is padded around the input image and between the adjacent two pixels of the input image, and in order to make the size of the output image twice that of the input, fill in extra 0 pixel on the right and upper sides. Then apply the convolution operation to the image behind the padding to get the result.

## 3. Implementation Details

### 3.1. Image Acquisition Mechanism

**Figure 8:**
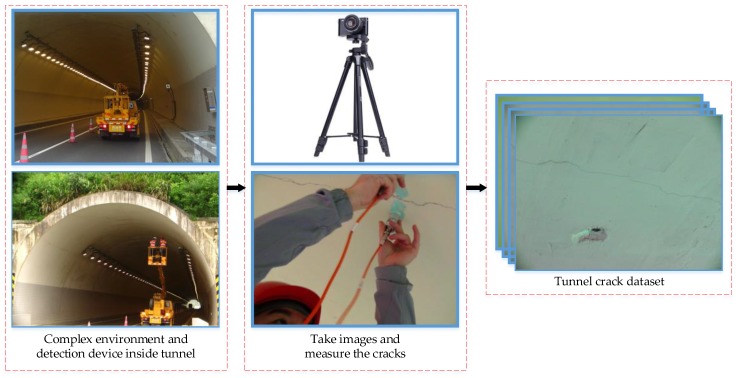
Schematic diagram of the tunnel crack image acquisition mechanism.

Since there is no publicly available tunnel crack dataset, the dataset we used was obtained through the crack acquisition mechanism. As different tunnels have various environments, we carried out image collection and detection from seven different tunnels. Figure 8 shows the interior of an ordinary tunnel and the process to take images. Capturing images of the tunnel requires the assistance of a climbing vehicle, which lifts the inspector close to the wall. The camera first needs to be placed on a tripod to keep the shot steady. During the progress, the inspector places the tripod about 50 cm from the wall to take images. The inspectors not only took photos of the cracks, but also measured the length and width of the cracks, so there are some images including cracks and handwriting in our dataset. Two hundred raw RGB images were taken by SONY DSC-WX700 (SONY, Wuxi, China) consumer-grade camera while the camera lens was kept perpendicular to the wall. The size of the original images we captured was 4896 × 3672 pixels, and were taken under different lighting conditions. The cracks in these images include longitudinal cracks, transverse cracks, oblique cracks, mesh cracks and so on. In addition to various types of cracks, the obtained crack images also contain various noises, such as electric wires, the calcimine peeling off and wall joints. 

### 3.2. Data Structure

The raw RGB images we obtained are 4896 × 3672, which is too large to be directly input into the network. To augment the dataset, we cut the original images into sub-images of 496×496 with a step of 248. The proposed method obtains the crack location in the image through semantic segmentation of the crack images, therefore, manual pixel-level labeling of training samples is required. Table 2 shows two original images with ground truth masks and the sub-images we obtained. All images from the training and testing sets were cut independently, and each raw image was cut into 234 sub-images. The cutting of crack pictures plays a certain role in the identification of cracks, because the cracks on the inner wall of the tunnel are very subtle and not easy to detect. After cutting the picture, the proportion of cracks in the picture with cracks becomes larger, making it easier to identify the cracks. A total of 200 original images were taken and cut into 46,800 sub-images. Since there are more non-crack images in these sub-images, in order to detect cracks and eliminate noise more effectively, a rotation operation is adopted to some sub-images with cracks and noises. The final dataset contains 60,000 sub-images, and we use 50,000 for training and 10,000 for testing to verify the effectiveness of the network.

### 3.3. Training Details

In order to optimize the parameters in the proposed U-CliqueNet, a number of optimization methods and hyper-parameters need to be set first. Parameters in convolutional layers and FC layers are initialized using the Xavier initialization [28,29]. When the stochastic gradient descent (SGD) method with momentum uses the well-designed stochastic initialization method, the performance of the trained model can be greatly improved [30], this study uses Mini-Batch Gradient Descent (MNGD) with 0.9 Nesterov momentum [31] to train our model. The batch size was n = 4 and the regularization weight was λ=0.0001. The initial learning rate is usually the single most important hyper-parameter, we should always make sure that it is tuned [32]. There are two structure parameters to set first [26], T is the sum of the layers of all the blocks, k represents the number of filters per layer in each block. The kernel size of convolution layers in all blocks are 3 ×3, and we use one-pixel padding to keep the size of the matrix the same before and after the convolution. The training progress was fulfilled on the computer which includes an Intel(R) Core(TM) i7-7800 CPU, 256-GB INTEL SSDPEKKR, and a NVIDIA GeForce 1080Ti GPU (Micro-Star, Beijing, China). The tensorflow framework based on Python 3.6 is used during the training progress of the proposed U-CliqueNet.

The loss function, also known as the error function, reflects the discrepancy between the predicted value and the ground truth. In the training process, the optimal solution needs to be obtained by constantly reducing the loss function. Therefore, the selection of different loss functions will have different effects on the training effect and what we use here is the cross entropy loss function. The parameters w and b are obtained by training the loss function. In our case, cross entropy is defined as:

(3) $$L\;=\;-\sum_{i=1}^{S^{2}}(y_{i}\log{\hat{y}}_{i}+\left(1-y_{i}\right)\log(1-{\hat{y}}_{i}),$$

where $y_{i}$ and $\hat{y_{i}}$ are the ground truth and prediction of the ith output unit, respectively; $s^{2}$ is the number of labels. In order to prevent the network from overfitting, our model adopts the method of weight attenuation to punish excessively large weight. The $L_{2}$ penalty is added into the loss function, so that the final loss function $L^{\prime }$ can be expressed as:

(4) $$L^{\prime }=L+\beta \frac{1}{2}\sum_{j}W_{j}^{2},$$

where L is the cross entropy function, β is the $L_{2}$ penalty factor and $W_{j}$ is the jth weight in the network, including weights in convolutional layers and fully connected layers. In the experiment, β is set to 0.0005 according to [33].

The problem of overfitting often occurs in the training progress of deep neural networks. Here a simple and effective technique is adopted to solve this problem, a dropout layer [34,35]. It is realized by randomly disconnecting the connections between neurons at a certain drop rate during training, in other words, we randomly set the weights of some hidden neurons to zero. It is applied after each convolution layer following with the probability of 0.2. 

### 3.4. Performance Evaluation Indicators 

There are three most commonly used evaluation indicators for the semantic segmentation, the pixel accuracy (PA), mean pixel accuracy (MPA) and mean intersection over union (MIoU) [36]. In the crack detection of this study, the crack is separated from the background, which is equivalent to binary classification. Table 3 lists four different kinds of identification states for each pixel of the input crack sub-image. True positive (TP) is summation of pixels that is truly recognized as crack. False positive (FP) represents the number of pixels that are recognized as crack, while they are not crack pixels. False negative (FN) is the number of pixels wrongly identified as non-cracks. True negative (TN) is the number of pixels correctly identified as non-cracks.

According to these four identification statuses for each pixel of the input crack image, the PA, MPA and MIoU, respectively, can be formulated as follows:

(5) $$\mathrm{PA}=\frac{TP+TN}{\mathrm{TP}+\mathrm{TN}+\mathrm{FP}+\mathrm{FN}}\;,$$

(6) $$\mathrm{MPA}=\frac{1}{2}\left(\frac{TP}{TP+FN}+\frac{TN}{TN+FP}\right),$$

(7) $$\mathrm{MIoU}=\frac{1}{2}\left(\frac{TP}{TP+FN+FP}+\frac{TN}{TN+FP+FN}\right),$$

In addition to the above three evaluation indicators, there are also three general indicators in crack detection, precision, recall and F1-score [19,25,37]. The precision represents the proportion of actual crack pixels in the predicted crack pixels, and the recall represents the proportion of correctly predicted crack pixels in the real crack pixels. The F1-score takes into account both model precision and recall, which can be regarded as a weighted average of precision and recall.

Precision indicates the proportion of the ground truth crack in the identification crack sets, while recall represents the percentage of the correctly recognized crack in the ground truth crack sets, they can be computed using TP, FP and FN as follows:

(8) $$\mathrm{Precision}=\frac{TP}{TP+FP},\;\mathrm{Recall}=\frac{TP}{TP+FN},$$

F1-score is a combination of precision and recall, which measures the average level of the algorithm, it is defined as:

(9) $$F1=\frac{2\times Precision\times Recall}{Precision+Recall},$$

The value of the F1-score ranges from 0 to 1 and is closer to 1 when the proportion of correctly detected images is higher. It is determined by the relative ratio of error and correct recognition in both the ground truth and recognition result sets.

## 4. Experimental Results

First, we selected the best learning rate through training and testing. Then we empirically demonstrate the effectiveness of the proposed U-CliqueNet on the tunnel crack datasets that we set up and compared with these most recent algorithms: FCN [23] proposed by Yang et al., U-net [25] proposed by Liu et al., Bang’s SegNet [38] and the MFCD [14] proposed by Li et al. In addition, skeleton extraction was carried out for the predicted binary image, next the area, length and width of the crack are calculated.

### 4.1. Selection of learning rate

**Figure 9:**
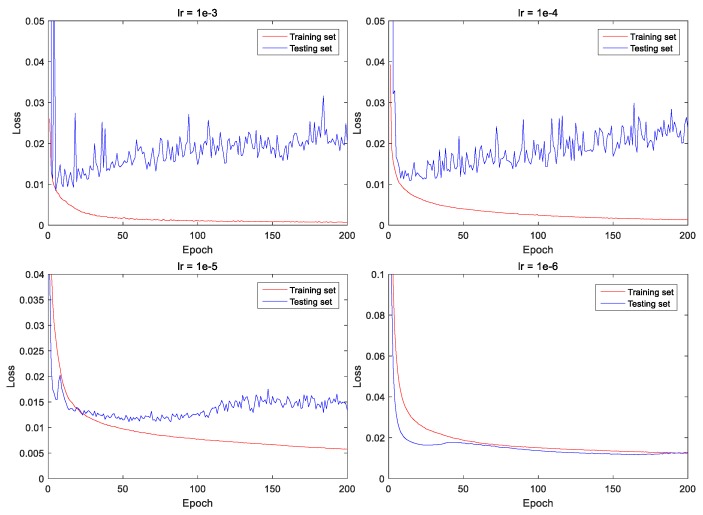
Loss function curves of training and validation datasets under four different learning rates.

Since the trained model has different performance under various learning rates, the selection of the learning rate plays a crucial role in the convergence of the loss function [30]. In order to find an appropriate initial learning rate, we conducted 200 iterations of training under four different learning rates. These four loss function curves are shown in Figure 9, where the solid red and blue lines represent the loss functions on the training set and the testing set, respectively. In fact, we only need to compare the results on the testing set, which is more convincing. When the learning rate is set to $10^{-3}$ or $10^{-4}$, the fluctuation of the loss function are relatively large, and the loss is between 0.015 and 0.025 after 200 iterations. Meanwhile, when the learning rate is set to $10^{-5}$ or $10^{-6}$, the loss on the testing set is stable at about 0.015 after 200 iterations. From the above comparison, it seems that the training loss is lower when the learning rate is set to $10^{-6}$. In order to verify whether the conclusion is right or wrong, we compare the models from the predicted results.

**Figure 10:**
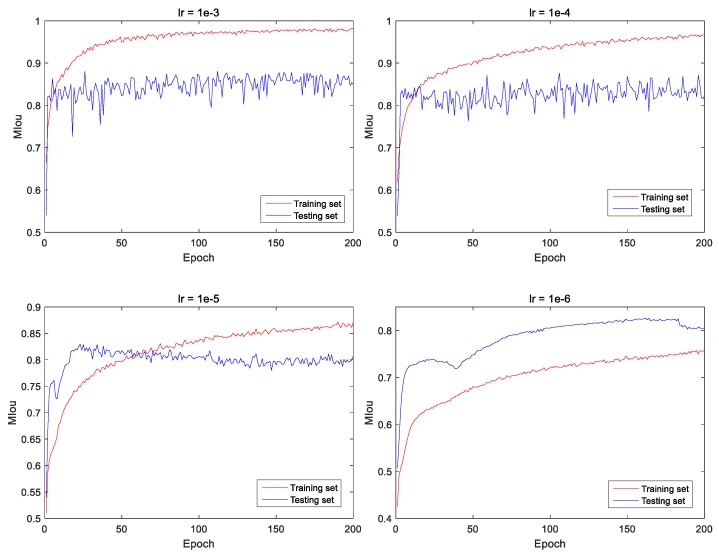
Mean intersection over union (MIoU) curves of training and validation datasets under four different learning rates.

The loss value does not fully reflect the effectiveness of the trained model, and another measure is the MIoU. Figure 10 shows the MIoU curves under four different learning rates, it can be seen that the MIoU on the testing set is the highest when the learning rate is set to $10^{-3}$ or $10^{-4}$. If the learning rate remains unchanged, the training process will fluctuate greatly when the learning rate is large. Therefore, the learning rate needs to be regulated during the training process. 

In this study, when the iteration reaches 100 times, the learning rate is reduced to 10% of the initial learning rate. Similarly, when the iteration reaches 150 times, the learning rate is reduced to 1% of the initial learning rate. 

**Figure 11:**
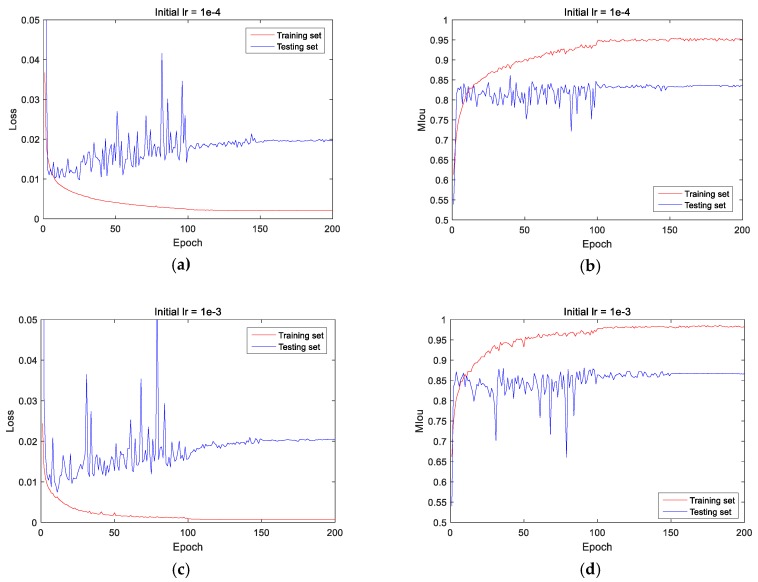
Loss and MIoU (mean intersection over union) curve after decreasing learning rate are adopted, (**a**,**b**) are the loss and MIoU curve when the initial learning rate is $10^{-4}$, (**c**,**d**) are the loss and MIoU curve when the initial learning rate is $10^{-3}$.

Figure 11 shows the loss function curve and MIoU curve under this learning rate setting method, it’s clear that the MIoU is much higher when the initial learning rate is set to $\;10^{-3}$ than $10^{-4}$. When the learning rate decreases after 100 epochs, the training range becomes smaller, and the training effect is significantly improved.

### 4.2. Comparison of Prediction Results

As described in Section 3.4, we use PA, MPA, MIoU, precision, recall and F1-score to evaluate a model. In this stage, we evaluate our method on the test dataset of tunnels that were never used in the training set and compare with the following recent methods: U-Net, FCN, SegNet and MFCD. Table 4 lists the performance of the five algorithms, it is clear that the MPA, MIoU and F1 of FCN are 88.25%, 83.91% and 78.54% respectively, which are all slightly lower than that of U-net. However, compared with the proposed U-CliqueNet, MPA, MIoU, precision and F1-score of U-Net are significantly lower, only PA and recall are relatively close. From the gaps in MIoU and accuracy, it can be seen that the performance of SegNet and MFCD is also good, but it is inferior to the above three methods. In MIoU’s evaluation, the calculated result is more representative of the accuracy of the algorithm, since cracks and backgrounds account for half of each. Therefore, compared with the other algorithms, the proposed algorithm has obvious advantages.

Among the six indicators, MIoU is the most important evaluation indicator, so we used the method of proportional hypothesis test to verify whether the MIoU of the proposed model is significantly larger than that of the other four methods. 

We randomly selected 20 images that did not belong to the training set and the test set, used the trained three models to predict and calculate the MIoU of each image. Table 5 lists the MIoU values predicted by these five methods for these randomly selected 20 images. 

Based on these data, we use hypothesis testing to determine whether the method in this paper is really superior to the others. Given a significance level of 0.1, the hypothesis test is performed with a 10% probability of making the second type of error.

Let the MIoU of FCN and U-CliqueNet be X and Y respectively, $X\sim N\left(\mu_{1},\sigma_{1}{}^{2}\right)$, $Y\sim N\left(\mu_{2},\sigma_{2}{}^{2}\right)$. $\left(X_{1},X_{2},\ldots ,X_{n1}\right)$ and $\left(Y_{1},Y_{2},\ldots ,Y_{n2}\right)$ are subsamples taken from X and Y, respectively. The mean value of subsamples is denoted as $\bar{X},\bar{\;Y}$, and the modified variance of subsamples is denoted as:

(10) $$S_{1}{}^{*2}=\frac{n_{1}}{n_{1}-1}S_{1}{}^{2}=0.00321,\;S_{2}{}^{*2}=\frac{n_{2}}{n_{2}-1}S_{2}{}^{2}=0.00322,$$

where $S_{1}{}^{2}$ and $S_{2}{}^{2}$ represent the variance of X and Y, respectively.

The hypotheses to be tested are:

(11) $$H_{0}:\;\mu_{1}=\mu_{2},\;H_{1}:\;\mu_{1}\neq \mu_{2},$$

Given $\mu_{1}=\mu_{2}$, select the test statistic:

(12) $$T=\;\frac{\bar{X}-\bar{\;Y}}{\sqrt{\left(n_{1}-1\right)S_{1}{}^{*2}+\left(n_{2}-1\right)S_{2}{}^{*2}}}\sqrt{\frac{n_{1}n_{2}\left(n_{1}+n_{2}-2\right)}{n_{1}+n_{2}}}\;\sim t\left(n_{1}+n_{2}-2\right),$$

When the significance level is 0.1, the rejection field is:

(13) $$\;\left|t\right|\geq \;t_{\alpha }\left(n_{1}+n_{2}-2\right)=t_{0.1}\left(38\right)=1.686,$$

If the calculated t is in the rejection field, the null hypothesis is rejected, that is, the performance of u-Cliquenet is considered to be significantly higher than that of FCN; otherwise, the null hypothesis is accepted. The data in Table 5 is substituted into Equation (12) for calculation, and the value of $t_{1}$ is −1.785. Because $t_{1}=\;-1.785<-1.686$, $H_{0}$ is rejected at the significance level of 0.10, and the MIoU of the proposed method is considered to be significantly higher than that of FCN. We also used the same method to conduct hypothesis testing analysis with U-net, SegNet and MFCD, and the results are $t_{2}=\;-1.693<-1.686$, $t_{3}=\;-2.607<-1.686$ and $t_{4}=\;-3.659<-1.686$. This means that the MIoU of the proposed method is also higher than that of the others. In addition, the p-values of the t-tests are calculated by MATLAB to determine whether it was significant or not. The calculation results are as follows: $p_{1}=0.0861$, $p_{2}=0.0987$, $p_{3}=0.0014$ and $p_{4}=0.0008$. The p-values of the four t-tests are all less than 0.10, therefore, we believe that our method is significantly better than FCN, U-net, SegNet and MFCD.

The internal environment of the tunnel is usually complex, with many patchwork joints, wires, etc. In order to verify whether the algorithm can separate cracks from images with noise similar to cracks, we detected many images with different noises. Table 6 lists five kinds of noises around cracks, which from left to right are the original sub-image, ground truth and prediction result. There is some handwriting in the first line of the image, which is very similar to the crack next to it. FCN and u-net wrongly identify handwriting as cracks, indicating that the robustness of the two algorithms is poor. Due to the improvement of the model and the enhancement of handwriting in training dataset, cracks and handwriting were perfectly distinguished from prediction results. On the second line, the crack is hard to detect because the wire has a high similarity to cracks in color and shape. There are also some spots around cracks such as in the image on the third line. The wall joint is another type of noise which is very similar to a crack, except that it is close to a straight line. Under the influence of so many interferences, the proposed model still correctly classifies these noises as background. Of course, there are other factors not taken into account, such as tunnel materials, but the above experimental analysis has shown that the proposed U-CliqueNet has great development potential in the field of crack segmentation.

The above experiments show that the proposed U-CliqueNet is excellent in reducing noise, and the specific analysis is described below. On the basis of U-net, the proposed model uses Clique block to replace the ordinary convolutional layer and reduces the number of down sampling. There are four convolution layers in each block, and the output of each layer is the input of all the other layers. The acquired features show more obvious differences between cracks, noises and background, which can be used to carry out crack segmentation more effectively. Different from FCN which only replaces the fully connected layer with the transposed convolution layer in the VGG16 network, this paper not only considers the feature learning in the downsampling process, but also considers the feature fusion in the upsampling process. Therefore, the proposed algorithm in this paper achieves better results in complex tunnel environment.

### 4.3. Crack Skeleton Extraction and Measurement

The purpose of crack semantic segmentation is not only to detect whether there are cracks in the image, but also to obtain the length or even width of cracks. Once the binary image of the crack is obtained, the length can be obtained by crack skeleton extraction. The skeleton of crack is extracted, that is, the crack of pixel level is converted to the width of single pixel level, which mainly reflects the morphology of the crack. In this paper, the median-axis skeleton extraction algorithm is used to remove the boundary of each crack, which has a good effect on the crack dataset in this paper. Table 7 lists the ground truth images and prediction images before and after extraction of crack skeleton, the single-pixel width cracks obtained after skeleton extraction has the same length as the original cracks. In the experiment, the skeleton extracted from the ground truth is taken as the real skeleton. The skeleton extraction is also conducted to the predict results by the proposed model, as shown in the last column, of the five images, four had a prediction error of less than 10%, while only one had a prediction error of more than 10%. By comparing the predicted results with the real results, it can be seen that the predicted crack skeleton is basically consistent with the real skeleton, which proves the effectiveness of the proposed algorithm and the skeleton algorithm.

After using the median-axis skeleton extraction algorithm for the prediction results, the area, length and average width of the crack can be calculated. The calculation method of crack length and width proposed in [23] is used, since the width of the crack skeleton is one pixel, the length of the crack can be obtained by calculating the number of all pixels in the skeleton images. The calculation formula of crack length L is as follows:

(14) $$L\;=\;\int_{c}^{}f\left(x,y\right)dl≅\sum^{\;}f\left(x,y\right)dl,\;$$

where f(x, y) is the geometric calibration index and dl represents the finite length of skeleton elements. In addition, the average width of cracks can be calculated as follows:

(15) $$\bar{W}=\frac{\int_{S}^{}f^{2}\left(x,y\right)dS}{L}≅\frac{\sum^{\;}f^{2}\left(x,y\right)dS}{\sum^{\;}f\left(x,y\right)dl},$$

where L and dS represents the crack length and finite area of crack elements, respectively.

**Figure 12:**
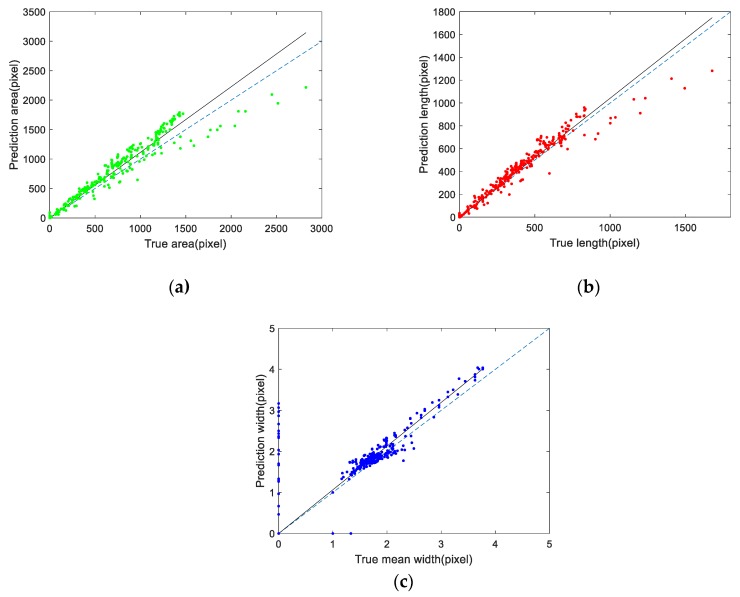
Scatter diagrams of crack measurement using U-CliqueNet: (**a**) crack area, (**b**) crack length, (**c**) crack mean width.

According to the above method, 500 sub-images were randomly selected from the test set for measurement. The area, length and mean width of cracks are measured in pixels. Figure 12 shows the scatter diagram of the real crack characteristics and the predicted crack characteristics by U-CliqueNet. The horizontal coordinate represents the ground truth value and the vertical coordinate represents the predicted value. After these corresponding points are obtained, we use linear regression to analyze the slope of these three histograms. The dashed lines in Figure 12a–c indicate that the predicted value is equal to the real value, while the solid lines represent the results after linear fitting. The slope of the histogram of crack area is 1.11, and its confidence interval is [1.097, 1.130]. The slopes of the histogram of length and width were 1.04 and 1.06, respectively, and the confidence intervals were [1.029, 1.055] and [1.036, 1.092] when the significance level was 0.95. The above research data show that the crack properties detected by this method are slightly larger than the real values. Furthermore, we use $R^{2}$ statistic, the F-statistic and its p-value to measure the effectiveness of linear fitting, the calculated results are shown in Table 8. The $R^{2}$ statistic and the p-value can reflect the fitting degree of linear regression. If the value of the $R^{2}$ statistic is larger, the fitting degree is higher, and if the p-value < 0.001, the fitting is effective. The results in Table 8 show that the results of linear regression fitting are highly effective. By comparing the real value with the test results, it is found that the predicted area and length are often larger than the real value, that’s because some non-crack pixels at the edge of the crack have features very similar to those of the crack. As can be seen from Figure 12b, the proposed method is very accurate in predicting crack length, with an average relative error of 9.65% and a maximum relative error of 27.77%. In the detection of crack mean width, the prediction results are stable near the real value, as shown in Figure 12c. For the calculation of mean crack width, only a few points are deviated significantly, the relative error of the other points is between −11.20% and 18.57%, while the average absolute error is 8.95%.

**Figure 13:**
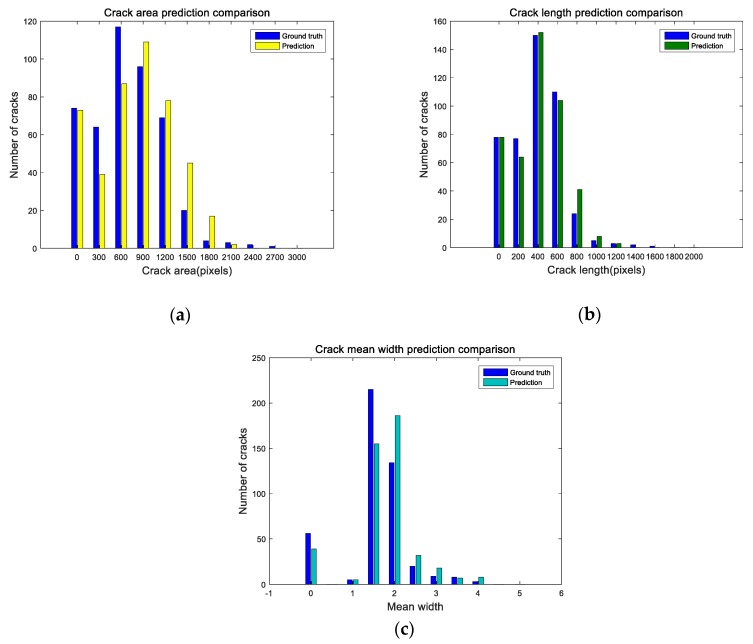
Histogram of crack measurement using U-CliqueNet: (**a**) crack area, (**b**) crack length, (**c**) crack mean width.

A histogram is an accurate graphical representation of the distribution of numerical data. Figure 13 shows the histogram of crack measurement, which clearly reflects the difference between the predicted results and the ground truth value. As can be seen from Figure 13a,b the proposed algorithm has a small error in predicting the crack area and width. When the crack width is less than 2 pixels, the predicted result is lower than the real value, while when the crack width is more than 2 pixels, the predicted result is slightly larger and the real value. The predicted crack length is basically consistent with the real value, as shown in Figure 13b. According to the above analysis, it can be concluded that the proposed U-CliqueNet is accurate enough for the detection of cracks.

## 5. Conclusions

In this study, a new deep learning framework called U-CliqueNet is proposed to detect tunnel cracks from images. It implements end-to-end crack prediction through a down-sampling path and an up-sampling path. Clique block and attention mechanism are introduced into U-net. There are two clique blocks in each path, where the input of each convolution layer is the input of other layers. The output of the clique block is the output of the last layer after two loops. A Sony DSC-WX700 camera was used to collect 200 raw images with a 4896 × 3672 pixels resolution. For the collected images, the corresponding labels are manually labeled at a pixel level. The raw images and labeled images are cut into sub-images of 496 × 496 pixels to increase the crack dataset and reduce the memory for each training session. The number of sub-images used for training and testing were 50,000 and 10,000, respectively. In order to find the optimal training model, the optimal learning rate of 0.0001 was obtained by setting different learning rates for experimental comparison. Based on the best training model, the U-CliqueNet achieves the highest MPA of 92.25%, MIoU of 86.96% and precision of 86.32%. Here, we use the method of hypothesis testing to analyze and conclude that the MIoU predicted by this model is significantly higher than that of FCN, U-net, SegNet and MFCD. The comparative study shows that the proposed method can provide better crack detection results than FCN and U-net in a dataset containing various noises. What’s more, skeleton extraction based on the segmented image can be used to calculate the crack area, length and average width, which greatly facilitates the actual crack detection. The relative measurement error of crack average width varies from −11.20% to 18.57%, which proves the reliability of the proposed method.

The proposed U-CliqueNet has a strong ability to detect internal cracks in tunnels, and with the expansion of datasets, the detection accuracy will be increasingly high. The nice thing about U-CliqueNet is that it is simple and efficient, requiring no extra processing. As long as the original images are input into the trained network, the prediction of cracks can be achieved. Although many kinds of complex crack images are collected in this study, they are still not completely satisfied in practice.

In future research, more images with more noises will be added to the existing tunnel dataset. Considering different damage inside the tunnel, multiple damage detection will be carried out.

## Figures and Tables

**Table 1:** Detailed properties of parameters for all layers.

Block	Layers	Output Size	Operator	Height	Width	Depth	No.
**Input**	Input	496×496×3	-	-	-	-	-
**Conv1**		496×496×36	conv	3	3	3	36
**CliqueBlock1**	$X_{1-4}^{\left(1\right)}$	496×496×36	conv	3	3	36	36
**TD1 (** **transition down)**		248×248×36	conv	1	1	36	36
		248×248×36	avp	2	2	-	-
**CliqueBlock2**	$X_{1-4}^{\left(2\right)}$	248×248×36	conv	3	3	36	36
**TD2**		124×124×36	conv	1	1	36	36
		124×124×36	avp	2	2	-	-
**CliqueBlock3**	$X_{1-4}^{\left(3\right)}$	124×124×36	conv	3	3	36	36
**TU1**		248×248×36	deconv	3	3	36	36
**CliqueBlock4**	$X_{1-4}^{\left(4\right)}$	248×248×36	conv	3	3	36	36
**TU2**		496×496×36	deconv	3	3	36	36
**CliqueBlock5**	$X_{1-4}^{\left(5\right)}$	496×496×36	conv	3	3	36	36
**Conv2**		496×496×36	conv	1	1	36	2
**Output**		496×496×2	-	-	-	-	-

**Table 2:** The original image and ground truth image is divided into sub-images.

Original Images	Ground Truth	Sub-Images
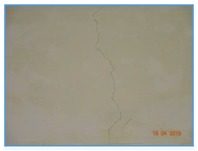	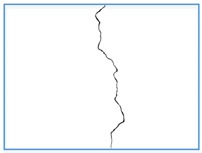	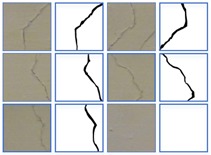
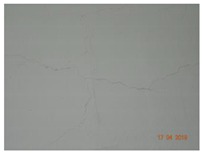	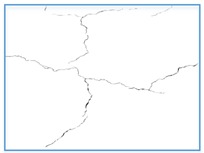	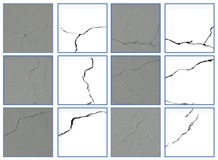

**Table 3:** Four identification statuses for each pixel of the input crack sub-image.

	Ground Truth	Crack (True)	Noncrack (False)
Prediction			
Crack (positive)		TP (True positive)	FP (False positive)
Noncrack (negative)		FN (False negative)	TN (True negative)

**Table 4:** Performance of these five algorithms: the fully convolutional networks (FCN), U-net, Encoder–decoder network (SegNet), the multi-scale fusion crack detection (MFCD) algorithm and the proposed U-CliqueNet.

Method	PA	MPA	MIoU (Mean Intersection over Union)	Precision	Recall	F1
FCN	0.9538	0.8825	0.8391	0.8269	0.7966	0.7854
U-net	0.9642	0.9059	0.8403	0.8457	0.8068	0.7967
SegNet	0.9385	0.8677	0.8050	0.7968	0.7495	0.7536
MFCD	0.9574	0.8850	0.7987	0.7937	0.8125	0.7808
U-CliqueNet	0.9661	0.9225	0.8696	0.8632	0.8028	0.8340

**Table 5:** The MIoU predicted by FCN, U-net, SegNet, MFCD and U-CliqueNet for randomly selected 20 images.

Image	FCN	U-net	SegNet	MFCD	U-CliqueNet
1.png	0.838	0.799	0.816	0.772	0.861
2.png	0.836	0.820	0.828	0.779	0.862
3.png	0.763	0.754	0.711	0.696	0.765
4.png	0.748	0.780	0.771	0.758	0.803
5.png	0.819	0.758	0.775	0.761	0.813
6.png	0.717	0.778	0.702	0.744	0.773
7.png	0.802	0.795	0.798	0.767	0.847
8.png	0.834	0.799	0.808	0.775	0.856
9.png	0.843	0.821	0.792	0.797	0.870
10.png	0.892	0.890	0.838	0.790	0.885
11.png	0.832	0.909	0.836	0.813	0.901
12.png	0.863	0.865	0.890	0.791	0.890
13.png	0.909	0.903	0.953	0.863	0.943
14.png	0.919	0.900	0.871	0.947	0.949
15.png	0.910	0.914	0.877	0.855	0.953
16.png	0.900	0.942	0.921	0.896	0.964
17.png	0.867	0.820	0.790	0.783	0.866
18.png	0.798	0.759	0.747	0.763	0.817
19.png	0.813	0.836	0.820	0.862	0.853
20.png	0.777	0.851	0.777	0.762	0.841
average	0.834	0.835	0.816	0.799	0.866
variance	0.00305	0.00361	0.00393	0.00328	0.00306

**Table 6:** Prediction result of cracks with different noises.

	Sub-images	Ground Truth	U-Clique Net	FCN	U-Net
**Crack with handwriting**	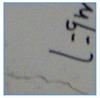	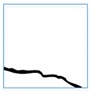	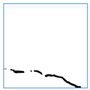	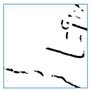	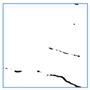
**Crack with** **wire**	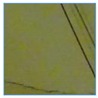	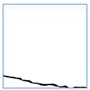	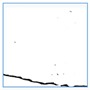	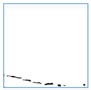	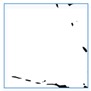
**Crack with** **spots**	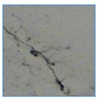	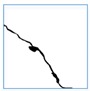	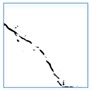	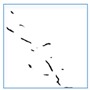	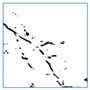
**Crack with** **wall joint**	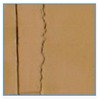	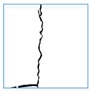	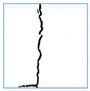	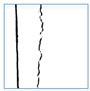	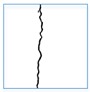
**Crack near** **the light**	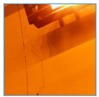	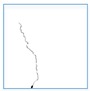	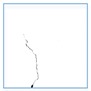	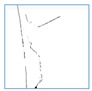	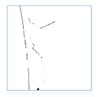

**Table 7:** Crack skeleton extraction of ground truth and prediction results.

	Ground Truth Masks	Ground Truth Skeleton	Prediction Masks	Prediction Skeleton
**Transverse** **crack**	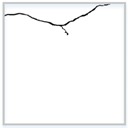	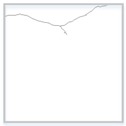	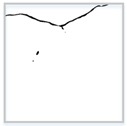	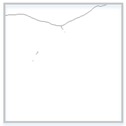
**Diagonal** **crack**	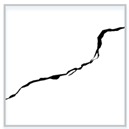	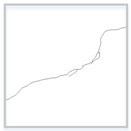	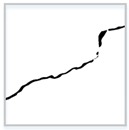	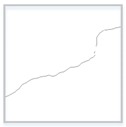
**Reticular** **crack**	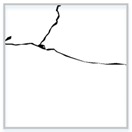	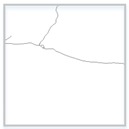	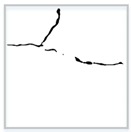	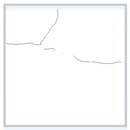
**Diagonal** **crack**	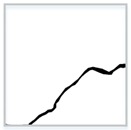	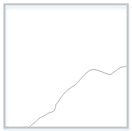	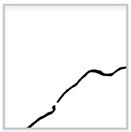	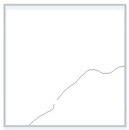
**Transverse** **crack**	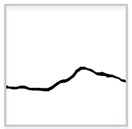	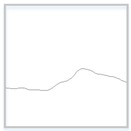	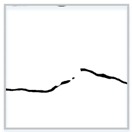	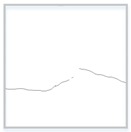

**Table 8:** Linear regression analysis of fracture measurement results.

	Area	Length	Mean Width
slope	1.11	1.04	1.06
confidence intervals	[1.097, 1.130]	[1.029, 1.055]	[1.036, 1.092]
$R^{2}$ statistic	0.922	0.943	0.706
F-statistic	100	743	531
p-value	<0.001	<0.001	<0.001
